# Contributions of a University Extension Programme in Special Care Dentistry to Education and Training of Undergraduate Students: A Qualitative Study

**DOI:** 10.1111/eje.13106

**Published:** 2025-04-27

**Authors:** Riéli Elis Schulz, Érica de Jesus, del Rosário Ruiz Nunez, Mariah Luz Lisboa, Ana Lúcia Schaefer Ferreira de Mello, Alessandra Rodrigues de Camargo

**Affiliations:** ^1^ Graduate Program in Dentistry Universidade Federal de Santa Catarina Florianópolis Brazil; ^2^ Hospital Dentistry Center, Polydoro Ernani de São Thiago University Hospital Universidade Federal de Santa Catarina Florianópolis Brazil

**Keywords:** dental care, dental care for disabled people, dental students, people with disabilities, qualitative research

## Abstract

**Background:**

People with disabilities (PWD) have poor oral health and difficulties in accessing dental care. The lack of skills, willingness, or confidence of dentists to treat PWD could contribute to this scenario.

**Objective:**

To understand the contributions of the Special Care Dentistry university extension programme to the professional and personal development of undergraduate students based on their perceptions.

**Study Design:**

Qualitative research was conducted with 12 undergraduate students who participated in the university extension programme, totalling 257 h of practice. Data was collected through a written questionnaire with open‐ended questions before and after their engagement in the programme activities. These data were analysed using the inductive content analysis technique, with coding and categorisation processes.

**Results:**

We considered two categories of contributions: professional and clinical experiences in dental care for PWD and the student teaching‐learning process. The first addressed the singularities of dental care for PWD, the apprehension of the student, and the need for humanised care. The second highlighted expanding professional opportunities, improving skills, and pursuing lifelong learning and lessons. In addition, the regular undergraduate curriculum added theoretical and clinical experiences.

**Conclusions:**

Students reported a significant improvement in confidence and safety for dental care for PWD by developing their critical thinking and clinical care skills.

## 1 Introduction

Over one billion people worldwide live with some form of disability, among which about 200 million experience considerable functional difficulties. Disability refers to any limitation or restriction that affects bodily functions or structures, impacting individuals identified as people with disabilities (PWD) [1]. In North America, approximately 56.7 million (1 in 5 citizens) are affected by physical, mental, behavioural, or emotional disability [2]. According to a national survey, 17.3 million people in Brazil have some form of disability [3]. PWD require access to specialised health care, health interventions, and/or specialised services or programmes [4].

Dental care represents one of the greatest unmet health needs of PWD [5]. The oral health of PWD is impaired by a higher prevalence of some oral diseases due to immune factors, financial reasons or even issues inherent to the disease itself, and these patients correspond to a significant portion of the population with difficult access to dental treatments [6]. In addition, PWD frequently encounter substantial barriers to accessing essential dental treatments, such as limited accessibility, comorbidities, communication difficulties, and challenges in maintaining proper oral hygiene at home [7]. Special Care Dentistry (SCD) assists people with genetic and/or acquired diseases, such as neurological motor disorders that can cause motor development delay and some neurological difficulties (e.g., cerebral palsy), genetic conditions (e.g., Down syndrome), chronic systemic diseases (e.g., diabetes mellitus, heart disease, systemic arterial hypertension), cancer and hematologic disorders (e.g., leukaemia, lymphoma), infectious diseases (e.g., HIV, hepatitis B or C), physical disability (e.g., paraplegia, hemiplegia), sensory disorders (e.g., hearing and visual impairment) and acquired diseases (e.g., rubella, tuberculosis) [8].

In south‐eastern Brazil, 62.5% of dental schools offer the SCD course, of which only about 70% are mandatory in the curriculum of the undergraduate course in Dentistry [9]. Although dental schools allow undergraduate students to assist this group, it seems insufficient to address dental care for PWD. Most SCD learning occurs through theoretical content courses and/or internships. However, whether different strategies focused on students' learning processes ensure high‐quality healthcare for PWD remains uncertain [10].

Improving access to dental care for PWD involves several strategies, such as disease prevention, dental care itself, and better clinical training of dentists and students [6]. Previous studies have demonstrated limited student competence in providing dental care for PWD upon graduation. A study with 295 dental students from five colleges in the United States of America (USA) on their preparation for treating patients with intellectual disabilities showed that about 60% of participants had little confidence in providing dental care for PWD. This can indicate inadequate preparation for treating PWD. The authors concluded that spending more time with these patients and experiencing practical teaching activities can improve students' understanding and ability to treat them [11].

Waldman and Perlman [5] discussed challenges in providing dental care for PWD in the USA, noting dentist reluctance due to insufficient training and low Medicaid reimbursement. The authors emphasised that adequate dental education and reimbursement policy changes are important measures to improve access. As many dentists are reluctant to treat PWD due to inadequate preparation, experience, and knowledge, the authors indicate that students should have more opportunities in dental school to provide high‐quality healthcare assistance [12].

Under the guidance of professors and tutors, extension programmes within undergraduate courses in dentistry in Brazil are recognised as extracurricular initiatives designed to enhance practical expertise, knowledge integration, and foundational skills necessary for future professionals. These initiatives are categorised as university extensions, representing an educational, cultural, and scientific process that closely intertwines teaching and research and connects university and society. Students can participate in extension programmes tailored to their personal or professional interests, often centred on primary and tertiary healthcare settings. The primary instructional approach revolves around experiential learning through professional practice, promoting meaningful interactions between students and society and developing a socially impactful scientific knowledge [13, 14, 15, 16].

As far as we know, no data were found in the literature reporting data on extension programmes focused on dental care for PWD. Therefore, the following research question was developed: What do undergraduate dental students perceive in their participation in immersive dental care for PWD in a university extension programme regarding its contributions to their professional and personal development? This study aimed to understand the contributions of the SCD extension programme to the professional and personal development of undergraduate dental students based on their perceptions.

## 2 Materials and Methods

This is an exploratory and descriptive study with a qualitative approach. It was approved by the research ethics committee (protocol number 984,051). It was designed and reported according to the Consolidated Criteria for Reporting Qualitative Research (COREQ) [17].

The sample was intentionally composed of 12 students who participated in the ‘University Extension Program of Outpatient Clinic of Dental Care for People with Disabilities’ at the Hospital Dentistry Centre of the University Hospital in southern Brazil. The university extension programme proposes an internship for undergraduate and graduate students in the tertiary care setting. It aims to develop skills and competencies in the outpatient dental care of patients diagnosed with intellectual disabilities through behaviour management techniques, emphasising communicative techniques and pharmacological management. The extension also includes visiting a surgical centre to monitor dental care under general anaesthesia.

This programme has been active since 2017. Students must have taken a 36‐h SCD theoretical course as a prerequisite for participation. The extension programme is eligible for annual university scholarships and has received a scholarship grant from its inception. To increase participation opportunities, all other students engage in the programme voluntarily. Activities are conducted weekly over an 18‐week cycle (totalling 257 h per academic year). Students need to undergo a project‐specific application and selection process to participate. Only actively enrolled undergraduate students at the university can participate since the programme is designed to complement their ongoing academic curriculum. Since its inception, the me has trained 48 undergraduate students in dentistry, four undergraduate students in speech‐language pathology and nine graduate students in dentistry (specialisation and doctoral degrees). In the last 6 years, the activities have assisted 219 patients.

The inclusion criteria for selecting participants were attending the last year of the undergraduate course in dentistry (ninth semester out of ten) and answering initial and final research questionnaires. Students were invited to voluntarily answer the questionnaires sent by the extracurricular programme coordinator and leading researcher from March to December 2018 by e‐mail. Data was collected through an open‐question form, answered in writing, individually and anonymously, by the student.

At the beginning of the programme, the questionnaire addressed expectations regarding the heterogeneity and concerns about dental care for PWD, and the rating scale for expectation/concern about working on the programme. At the end of the extension programme, the questionnaire addressed the experience acquired, the positive aspects experienced, and the comfort and concern levels when performing dental care for PWD during the programme (Supporting Information).

The inductive content analysis technique was adopted for data analysis [17]. The textual analysis was carried out in three stages: Pre‐analysis, data exploration, and result analysis, with inference and interpretation. In the pre‐analysis stage, two researchers conducted the ‘floating reading’ of the raw textual answers for data organisation. Then, the same two researchers analysed the relevant data to answer the research question. Also, in this stage, the analytical categories for data exploration were defined [18, 19]. The data exploration stage coded and categorised the textual content. Coding transforms raw data (units of record or meaning) into concepts. Categorisation classifies codes by similarity or differentiation. Data was analysed and grouped according to the nature of the information. Finally, in the result analysis stage, inferences and interpretations were performed to qualitatively analyse the categories and their interrelationships.

## 3 Results

Twelve students participated in this study, nine of whom were female. Their mean age was 23 years (ranging from 22 to 27 years), and they had completed approximately four and a half years of academic training.

We structured the results into two overarching categories: (1) professional and clinical experiences in dental care for PWD and (2) student teaching–learning process. We identified these categories through the systematic coding of students' responses, highlighting key aspects of their experiences in the extension programme. The first category encompassed the challenges, emotional responses and professional and clinical skills acquired to provide dental care for PWD. The second category highlighted the broader impacts of the extension programme on students' professional and personal development. We further divided each category into sub‐categories to represent specific aspects of student experiences about the contributions of the university extension programme (Tables 1 and 2).

**TABLE 1:** Contributions of a university extension programme for special care in dentistry for education and training of undergraduate students: Subcategories and respective codes of the **c**ategories: Professional and clinical experiences in dental care for PWD.

Categories	Codes
Initial exposure to dental care for PWD	To know how to act professionally To learn from the difficulties To develop technical–clinical skills and gain agility in clinical care The relationship and care with the patient who presents unique conditions To put theoretical knowledge into practice To find the best way to manage each patient The need for multidisciplinary teamwork To acquire security and confidence to care for PWD To perform procedures in a stressful environment and work under pressure To work under constant patient movement To work with a relatively shorter consultation time The patient sedation procedure To predict where the patient may feel pain even though they are not talking or reacting PWD can be assisted in a conventional dental office and do not always require a special hospital environment To provide dental care when the patient is not collaborative Losing the fear of caring for PWD Losing fear of using ‘unusual’ medications
Managing anxiety and emotional challenges	Fear of making a mistake that causes pain, suffering, or hurt To think they did the best for that situation To have support from the hospital professionals Concerns related to technique execution Concerns for the patient's health Need to improve theoretical basis To feel safe in the face of a patient at risk Concern about the relationship and bond created with the patient To control the anxiety in the face of unexpected situations Communication, understanding what the patient is feeling The need to immobilise the patient Fear of taking too long in dental procedures Difficulty in working under pressure Not having the necessary motor skills
Developing a patient‐centred approach and humanised care	Dentistry can make a difference/change people's lives To promote health and quality of life of the PWD Improving the quality of the service with a more humanised dental care To meet the patients' expectations To create a link of trust with PWD and their families To develop sensitivity toward PWD and their families To understand what the patient is feeling Each patient is unique The desire to do something for PWD and their family To develop a human look toward PWD

**TABLE 2:** Contributions of a university extension programme for special care in dentistry for education and training of undergraduate students: Subcategories and respective codes of the categories: The student teaching–learning process.

Categories	Codes
Expanding professional opportunities	In most Brazilian cities, there are no experts in this area The demand exists but is not being met by Brazilian dentists There is a lack of professionals who feel capable of assisting these people To work as a dentist in a hospital To become an expert in SCD later To expand the vision and knowledge about the PWD To improve access and agility in dental services for the PWD To deal with the patient's family To work as a team To classify the degree of dental care difficulty At least try to assist these patients before referring them to specialised care
Integrating extension activities into academic training	Put knowledge into practice First contact with the PWD The freedom to care for PWD Unique opportunity and challenge to get in touch with PWD To be able to communicate with PWD and their families To do something different from the curricular dental care To work with several professionals from different areas To learn to work as a team Living with completely different realities To improve the ability to read scientific articles To recognise when they are unable to assist alone and need to refer the PWD to specialised dental care Experiencing something different from what is offered by the regular undergraduate curriculum The level of concern to deal with PWD has diminished
Personal and professional development	To be more human and mature To grow as a human being To understand the life story of PWD and families To do good To understand that will face many difficulties To realise the difficulties and victories of the patients' families Fear is just a detail because, during dental care, it does not exist To realise that they are more capable than they had imagined To feel confident To leave the ‘comfort zone’ To bring a significant professional gain Many barriers were broken, and many paradigms and fears were undone

### 3.1 Professional and Clinical Experiences in Dental Care for PWD

#### 3.1.1 Initial Exposure to Dental Care for PWD

This sub‐category describes the students' first direct contact with dental care for PWD. Students emphasised that this experience was fundamental in their professional training, as it enabled them to develop technical–clinical skills in an environment similar to real‐world dental practice. The hospital environment introduced students to the complexities of working under time‐sensitive and high‐pressure conditions, enhancing their agility and adaptability. The experience significantly differed from their previous work in undergraduate clinics, where patient interactions were more predictable. Moreover, students highlighted the importance of applying knowledge from the SCD theoretical course to practical situations, reinforcing their learning and skill acquisition.

#### 3.1.2 Managing Anxiety and Emotional Challenges

Students initially experienced anxiety and uncertainty when treating PWD, primarily due to concerns about causing discomfort or inadequate communication. Many students expressed apprehensions regarding technical aspects, such as performing restorations with aesthetic concerns, managing sedation, anaesthetising patients with anatomical variations, and maintaining efficiency while ensuring patient safety. The lack of prior exposure to dental care for PWD exacerbated these fears. However, the structured support of the hospital setting, faculty supervision, and collaboration with a multidisciplinary team provided reassurance, enabling students to transition from uncertainty to confidence in their abilities.

#### 3.1.3 Developing a Patient‐Centred Approach and Humanised Care

Students reflected on the importance of humanising dental care for PWD, emphasising that dentistry should address oral health and enhance the overall well‐being and quality of life of patients. Through their experiences in the extension programme, students developed a deeper appreciation for patient individuality, understanding the significance of medical history, family context, and specific needs when delivering care. The realisation that many PWD had been previously underserved reinforced the importance of establishing trust and fostering long‐term patient‐provider relationships. These experiences contributed to a broader and more compassionate perspective on dental practice.

### 3.2 Student Teaching–Learning Process

#### 3.2.1 Expanding Professional Opportunities

This sub‐category highlights the impact of the extension programme on students' professional goals and career paths. Students recognised the increasing demand for trained practitioners to serve PWD, given the limited number of specialised professionals available in the region. The programme exposed them to unique clinical challenges, fostering critical thinking and adaptability. In addition, hands‐on experience and theoretical learning from scientific literature enhanced their ability to assess and refer to cases appropriately. The programme also sparked interest in pursuing a specialisation in PWD dentistry among several students who recognised the societal need for this expertise.

#### 3.2.2 Integrating Extension Activities Into Academic Training

This sub‐category explores how the extension programme complemented and enriched undergraduate education. Students indicated that the programme bridges the gap between theory and practice by reinforcing classroom knowledge through clinical application. Faculty guidance encouraged autonomy in decision‐making, prompting students to refine their clinical reasoning and problem‐solving skills. Furthermore, students encountered communication barriers and patient management challenges not typically addressed in standard undergraduate curricula. Through multidisciplinary collaboration, they learned to navigate these obstacles, ultimately contributing to patient care and their academic growth. The extension programme also increased treatment of PWD, extending the benefits beyond student learning to positively impact patient outcomes.

#### 3.2.3 Personal and Professional Development

Students emphasised the broader, long‐term effects of participating in the extension programme. Beyond technical skills, they reported developing a sense of resilience, empathy, and responsibility toward underserved populations. The exposure to the patients' life stories and daily struggles reshaped their perspectives, reinforcing the value of compassion in dental practice. The programme provided a structured environment for students to overcome their initial fears and insecurities when sharing these challenges. The transition from uncertainty to confidence was a recurring topic, with students recognising that the experience introduced a lasting commitment to patient‐centred care. Ultimately, they left the programme feeling more prepared to serve PWD in their professional careers, recognising the profound impact of their contributions.

In summary, the extension programme contributed to students' professional and personal development. The results demonstrated how direct exposure to dental care for PWD fosters technical competence, emotional resilience, and commitment to humanised dentistry. The programme activities expanded students' professional opportunities and reinforced the importance of patient‐centred care, addressing a critical need in the SCD field.

## 4 Discussion

Our results showed that the first contact with the specificities of dental care for PWD generated anxiety in undergraduate dental students. As the area presents singularities in managing each patient, the students felt anxiety about practising the knowledge acquired in theoretical classes during the undergraduate course. Attending lectures did not guarantee their confidence in clinical care since undergraduate students are still refining procedural techniques.

For Lage et al. [20], when the course requires students to develop practical skills, the methodology of purely expository classes, in which the professor is seen as the ‘holder’ of knowledge, is not as effective as active methodologies that encourage students to practice their skills. Therefore, the extension programme was essential for students to bridge the gap between theoretical classes and clinical activities involved in dental care for PWD.

Students identified clinical courses, practical classes, and integrated multidisciplinary clinics as the most significant components of their learning process [15]. However, they also recognised the value of introductory courses, supervised internships, participation in research and extension programmes, and their undergraduate thesis for their overall education [15].

Our findings indicated that undergraduate dental students experienced anxiety when dealing with PWD. Their limited or no experience in providing dental care for PWD raised concerns about working under pressure, managing constant patient movement, navigating potential anatomical variations, handling clinical time, conducting sedation interventions, ensuring patient acceptance of treatment, and dealing with a stressful hospital environment. In addition, establishing relationships and effective communication with patients is commonly challenging or even non‐existent. All these anxieties led to their primary concern: The patient's overall health since they aim to offer the best possible dental treatment while protecting patients from pain and/or suffering.

According to Perusini et al. [21], a lack of experience in managing PWD affected 70% of the 92 students interviewed from the Faculty of Medicine and Dentistry in Toronto, Canada. In contrast, only 7% reported an excellent experience, while just 6% indicated a great experience. This suggests that 46% of students do not feel comfortable providing dental care for PWD. Despite the progress in treating PWD, the curricula change slowly, particularly considering the significant demographic changes in this population, which may not be sufficient to meet the increasing demand. Subar et al. [22] emphasised that developing dental skills is essential for dental care for PWD. Therefore, universities and colleges are responsible for ensuring that undergraduate training remains up‐to‐date to address the increasing demands of this population.

Undergraduate courses in dentistry are responsible for theoretically and technically teaching dental care for PWD. They offer the supervision of professors and/or monitors trained for this activity. Clemetson et al. [23] showed that 80% of 54 schools in the USA and Puerto Rico answered ‘definitely yes’ or ‘probably yes’ to the importance of spending more time teaching the treatment of PWD. We believe that practical SCD for PWD should not be an extracurricular programme. The curriculum already includes theoretical discipline, but should implement new practical approaches to bridge the gap between university and clinical practice. If this does not occur, professors committed to addressing the issue should create alternatives, such as extracurricular extension programmes.

The technique used in clinical procedures was one of the most anxiety‐provoking factors among the students. However, we believe that after completing the extension programme, the students felt capable of developing humanised dentistry. During the consultations, the students could have contact with the life stories of their patients, family members, and/or caregivers. This intimate contact with the stories of difficulties in accessing health services, the dental treatments neglected by unprepared professionals and the uncertainty of finding a service capable of meeting their dental demands was softened by stories of courage, struggle and, above all, perseverance of patients, family members and/or caregivers to overcome significant barriers experienced by PWD. Thus, the students developed a humanised view of the PWD, progressing in executing patient‐centred and humanised care. The experience through the extension programme favoured professional growth with great human value.

This study also identified that the experiences in the programme changed students' views about their future profession, particularly in the PWD area. The extension programme was essential for connecting with this vulnerable population and adding knowledge regarding management, attention, agility, and understanding of their needs. The PWD demand some singular approaches and procedures in their care. However, during the learning process offered by the extension programme, the students realised they could resolve most clinical situations. There was no need for deep and specialised training before caring for these patients. Still, they had the opportunity to become closer and more sensitive during the undergraduate course in dentistry. This perception brought confidence and a desire to provide dental care for PWD after graduation in clinical practice scenarios outside the university.

Students who participated in clinical activities with PWD tend to increase their availability to care for this population. The hypothesis addressed in this study and confirmed by Perusini et al. [21] and Chavez et al. [12] demonstrates that the levels of comfort and desire for treatment will continue to rise with the encouragement of extracurricular activities, in which students are the protagonists, and may last in their professional life after graduation.

These supervised extension programmes are initiatives of Brazilian Universities. Based on the experience in these programmes and our results, we observe that these programmes provide more security to students since they face real and practical situations of PWD care. Another successful example of these extension programmes is the PET (in English, Tutorial Education Program) [16]. PET is a Brazilian government initiative aimed at promoting the academic development of undergraduate students by encouraging activities in teaching, research, and extension. It aims to stimulate high‐quality academic education, foster integration among undergraduate, graduate, and elementary schools, encourage student engagement in extension, research, and teaching activities, and enhance the quality of undergraduate student education.

Students with more opportunities to assist PWD during their academic training reported assisting significantly more PWD in their work routine as trained dentists [12]. Therefore, the extension programme ‘Ambulatory of Dental Care for People with Disabilities’ contributed jointly to the professional and personal scope of the undergraduate student. This contribution was possible due to the previous SCD theoretical course, the discussion of scientific articles during the programme and the clinical experiences and direct contact with patients from the first day. Professors, monitors trained in the area, and a multidisciplinary team supervised the services provided by the extension programme. This knowledge enriched the undergraduate course experience and broadened the students' horizons in their final year of college. The students felt prepared to solve most cases by understanding the importance of quality dental care for PWD. This enables them to be future professionals who will avoid unnecessary referrals and solve, perhaps, essential problems at the primary health care level.

Another result was the significant contribution of the extension programme to the students' lives. The students reported that the programme enriched them both in technique and as human beings since many paradigms and fears were broken at the beginning of the programme. The care situations challenged the students to leave their comfort zones, contributing to their growth. By developing self‐confidence, they demonstrated greater capability in their clinical conduct than they expected, overcoming fear during the programme. Assisting more complex cases outside the university environment should receive encouragement, empathising with the patient, family members, and/or caregivers, and considering their life stories. Even though it was a short experience, it brought significant professional development.

This study is limited by the small sample size of 12 responses to a non‐mandatory questionnaire. This limited participation may mainly represent those students with a greater interest in the field. Thus, it potentially directed results toward a more positive perspective on the extension programme. Those who participated may have a more favourable view than non‐respondents. Furthermore, as the programme is not a curricular requirement, it tends to attract students interested in this field. Initial interest was developed during the mandatory SCD theoretical course. Despite these limitations, the findings offer valuable insights into students' perspectives gained through practical experience, emphasising the need for further research with larger sample sizes and more comprehensive tools. Additionally, this raises important questions regarding the effectiveness of undergraduate programmes in preparing students to treat PWD, suggesting that this care should be integrated into the core curriculum rather than limited to extracurricular activities.

## 5 Conclusions

Student participation in the programme added theoretical and clinical experience to the regular curriculum. Improvements were evident in acquiring confidence and comfort in dental practice. In addition, students reported developing their critical view of PWD and increasing their skills for treating PWD after graduation. They greatly benefited from the active teaching dynamic observed in the extracurricular activities. University programmes focused on the PWD population could also minimise inappropriate referrals to specialised dental services and increase alternative dental treatment approaches based on the knowledge and skills acquired during the undergraduate course in dentistry.

## Author Contributions

Érica de Jesus, del Rosário Ruiz Nunez, Ana Lúcia Schaefer Ferreira de Mello, and Alessandra Rodrigues de Camargo: Have made substantial contributions to conception and design, or acquisition of data, or analysis and interpretation of data; and Riéli Elis Schulz: Been involved in drafting the manuscript or revising it critically for important intellectual content; and Riéli Elis Schulz, Érica de Jesus, del Rosário Ruiz Nunez, Mariah Luz Lisboa, Ana Lúcia Schaefer Ferreira de Mello, and Alessandra Rodrigues de Camargo: Given final approval of the version to be published. Each author should have participated sufficiently in the work to take public responsibility for appropriate portions of the content; and Riéli Elis Schulz, Mariah Luz Lisboa, Ana Lúcia Schaefer Ferreira de Mello, and Alessandra Rodrigues de Camargo: Agreed to be accountable for all aspects of the work in ensuring that questions related to the accuracy or integrity of any part of the work are appropriately investigated and resolved.

## Ethics Statement

This study was performed in line with the principles of the Declaration of Helsinki. Approved by the research ethics committee with opinion number 984051.

## Consent

The authors affirm that human research participants provided informed consent for publication of the data. Informed consent was obtained from all individual participants included in the study.

## Conflicts of Interest

The authors declare no conflicts of interest.

## Supporting information


Appendix S1.

## Data Availability

The data that support the findings of this study are available on request from the corresponding author. The data are not publicly available due to privacy or ethical restrictions.
