# Supplementary material for: Contributions of a University Extension Programme in Special Care Dentistry to Education and Training of Undergraduate Students: A Qualitative Study
Source: Eur J Dent Educ. 2025 Apr 27;30(1):189–96. doi: 10.1111/eje.13106 (PMC12834537; doi:10.1111/eje.13106)
Supplement: Supplementary file 1 — Appendix S1. [file EJE-30-189-s001.docx]

**SUPPLEMENTARY FILES**

**Pre-Extension Project Questionnaire**

1 - What are your expectations regarding the care of people with disabilities (PWD), considering the heterogeneity of neuropsychomotor disorders?

2 - Please list your concerns regarding the dental care provided in the project.

3 - Can you rate the level of your expectations/concerns?

( ) none; ( ) low; ( ) moderate; ( ) high.

**Post-Extension Project Questionnaire**

1 - Describe the experience gained from the dental care provided by the extension project.

2 - Detail the positive aspects of the experience gained in your education.

3 - Describe your level of comfort and concern regarding the care of PWD with neuropsychomotor disorders after participating in the project.
